# MicroRNA-223 Attenuates Stretch-Injury-Induced Apoptosis in Brain Microvascular Endothelial Cells by Regulating RhoB Expression

**DOI:** 10.3390/brainsci12091157

**Published:** 2022-08-30

**Authors:** Yingliang Liu, Wenjing Li, Yingxiu Liu, Yang Jiang, Yida Wang, Zhiming Xu, Daming Cui, Liang Gao

**Affiliations:** 1 Department of Neurosurgery, Shanghai Tenth People’s Hospital, Tongji University School of Medicine, Shanghai 200072, China; 2 Shanghai Institute of Cardiovascular Diseases, Zhongshan Hospital, Fudan University, Shanghai 200032, China; 3 Department of Neurology, Zibo Ninth People’s Hospital, Zibo 256400, China; 4 Department of Neurosurgery, Shanghai Jiao Tong University Affiliated Sixth People’s Hospital, Shanghai 200233, China

**Keywords:** miR-223, RhoB, apoptosis, TBI, SI, BMEC

## Abstract

MiR-223 is a miRNA with important functions in apoptosis, carcinogenesis, and inflammation, and it was demonstrated to be over-expressed in brain tissue after traumatic brain injury (TBI). However, few studies have focused on its role in protecting brain microvascular endothelial cells (BMECs). This study evaluated the protective effect of miR-223 on BMECs after stretch injury (SI). bEnd.3 cells (BMECs of mouse) were transfected with overexpressing and blocking lentivirus of miR-223, then were subjected to SI. After immunofluorescence assay, it was demonstrated that miR-223 overexpression significantly rescued the SI-induced loss of ZO-1 (Zonula Occludens 1, tight junction protein) (*p* < 0.01), while miR-223 blocking exacerbated the loss of ZO-1 (*p* < 0.05). Flow cytometry confirmed a significant increase in the proportion of apoptotic bEnd.3 cells after SI, and miR-223 overexpression reduced this proportion (*p* < 0.001). The result of Western blot revealed that miR-223 overexpression significantly reduced the expression of cleaved caspase-3 (cl-caspase 3) (*p* < 0.05) and RhoB (*p* < 0.01), while miR-223 blocking increased the expression of these proteins (*p* < 0.05, *p* < 0.001). Additionally, knockdown of RhoB significantly reduced the expression of cl-caspase 3 (*p* < 0.001). These findings suggested that miR-223 can alleviate SI-induced apoptosis of BMECs, and this anti-apoptotic effect is at least partially achieved by inhibiting the expression of RhoB. Moreover, miR-223 may play a role in maintaining the integrity of BBB during TBI.

## 1. Introduction

MicroRNAs (miRs) are small non-coding RNAs that contain 20–25 nucleotides. They can negatively regulate the expression of messenger RNAs (mRNAs) [1]. In the past few decades, researchers identified miRs as major gene regulators. As reported, one-third of gene and protein expression is regulated by miRs. As a well-known miRNA, miR-223 plays an important role in cancer, infection, and the immune system [2], and the regulatory function of miR-223 on cell apoptosis, proliferation, and differentiation has been confirmed by many articles [3,4]. In recent years, miR-223 has been found to play an important role in the repair of brain damage caused by the febrile seizure and intracerebral hemorrhage [5,6]. Moreover, in previous studies, microarray and RT qPCR analysis showed that the expression of miR-223 was significantly increased in brain tissue of rats after TBI [7,8,9], but few studies have focused on the role of miR-223 in protecting of blood–brain barrier (BBB) and brain microvascular endothelial cells (BMECs).

The Rho (Rashomologous) protein family is a member of the Ras superfamily of medium molecular weight G proteins, which is a GTP-binding protein with a relative molecular weight of 20,000–30,000. RhoB, a member of the Rho subfamily, is a unique molecule in the Rho family that plays a role in intracellular localization, regulation, and function, etc. It is considered a negative regulatory gene of tumors. In many tumors, RhoB can induce apoptosis in tumor cells by activating the internal apoptotic cascade [10]. Based on this, Rhob has become an important target for regulating apoptosis in multiple cells and has been further studied.

BMECs are the main cells of cerebrovascular, and they build the vascular wall through tight junctions between cells, forming a barrier between brain blood and cerebrospinal fluid (CSF). It is also the most important part of the BBB, which can effectively inhibit the extravasation of intravascular substances and transport various necessary nutrients between blood and the brain so as to maintain the dynamic balance of the CNS microenvironment. Apoptosis and necrosis of BMECs often lead to damage to BBB integrity, which causes not only vasogenic brain edema but also brain tissue damage [11,12]. It is well known that BBB damage is an important part of many pathophysiological processes, such as TBI, intracerebral hemorrhage, cerebral ischemia, subarachnoid hemorrhage (SAH), and spinal cord injury. The BBB damage also increases the mortality and disability of patients [13,14]. However, studies on the relationship between miR-223, BMECs, and BBB integrity are still lacking. In this study, we demonstrated the inhibitory effect of miR-223 on apoptosis in BMECs and explored the protective role of miR-223 in maintaining the integrity of BBB.

## 2. Method

### 2.1. Endothelial Cells Culture

The protective effect and mechanism of miR-223 on BMECs were investigated by culturing bEnd.3 cells (mouse microvascular endothelial cell line). The cells were purchased from American Tupe Culture Collection (ATCC). Cells were cultured in DMEM medium (Gibco Laboratories, Grand Island, NY, USA) and supplemented with 10% fetal bovine serum (FBS) and 100 μg/mL streptomycin and penicillin. BEnd.3 cells were maintained in a sterile incubator infused with a gas mixture containing 5% CO_2_. For the experiment, cells were seeded on collagen-coated cover-slips or BioFlex elastic membrane supports (Flexcell International Corp, Burlington, NC, USA)) at a density of (0.14–0.6) × 105/cm^2^.

### 2.2. Mechanical Cell Injury

Stretch-induced cell injury is an in vitro injury model which is used to simulate the cell injury during TBI. It has become the most recognized cell damage mode by researchers, and its reliability has been verified many times in our previous experiments [15,16] and also by other groups [17]. BEnd.3 cells were grown to confluence in BioFlex six-well culture plates with collagen-coated Silastic membranes (Flexcell International Corp, Burlington, NC, USA), and the cells in different groups were transfected into miR-223, miR-223-block, or miR-control. After sufficient preparation, a Cell Injury Controller II system (Virginia Commonwealth University, Richmond, VA, USA) was used to generate biaxial stretch on the cells in the plate, delivering a 50 ms burst of nitrogen gas that produced a downward deformation of the Silastic membrane and adherent cells. In our study, a 7.5 mm membrane deformation was used to cause damage to cells, which was used to simulate mechanical stress damage on the surface of the human brain. After 24 h of continuous incubation, cells were ready for examination.

### 2.3. Cell Transfection

In order to study the role of miR-223 in apoptosis of bEnd.3 cells, cells were transfected by lentivirus. Lentivirus of miR-223 (miR-223 overexpression sequence) or miR-223-block (miR-223 response sequence) and their corresponding miR control (NC) or anti-miR were purchased from Shanghai Han heng Bio Co., Ltd. (Shanghai, China) 2 μL lentivirus was added into each well of bEnd.3 cells on a six-well plate. After 12 h of transfection, the medium was changed. A fluorescence microscope was used to observe the transfection rate of bEnd.3 cells after cell passage, If the rate was over 80%, the cells could be used for the experiment. SiRNA to RhoB, 5′-CCGUCUUCGAGAACUAUGU-3′ (20 nM) was transfected into bEnd.3 cells by using Lipofectamine LTX reagent according to the manufacturer’s protocol. After 24 h, the effects of knockdown on cell survival and signal transduction were assessed. The level of gene knockdown by siRNA was verified by RT-PCR.

### 2.4. Immunostaining

BEnd.3 cells were stained with ZO-1 (Invitrogen, Carlsbad, CA, USA) to evaluate the damage to tight junction protein in the miR-223 or miR-223-block group after stretch injury, and the main steps were as described in the previous experiments [18,19]. Briefly, cover-slips with bEnd.3 cells were blocked with 10% fetal bovine serum for 1 h and then incubated with antibodies (1:(100–200) dilution) overnight at 4 °C. After washing, appropriate secondary antibodies were used to incubate the cover-slips for 1 h at 37 °C. Finally, these slips were examined through a confocal microscope (Leica), and the average fluorescent area of ZO-1 in bEnd.3 cells was assessed using Image J software (NIH, Bethesda, MD, USA).

### 2.5. Relative Real-Time Polymerase Chain Reaction

Cultured bEnd.3 cells were collected, and total RNA and microRNA were isolated by TRIzol Reagent (Invitrogen, Carlsbad, CA, USA) and miRNeasy Kit (Qiagen, Germantown, MD, USA). The RNA concentration was measured by a nanodrop 1000 micro spectrophotometer (Thermo, Wilmingtong, DE, USA). The Od260/od280 values between 1.8 and 2.0 were qualified samples that would be used for polymerase chain reaction (PCR) detection. One Step SYBR^®^ (Takara bio, Shiga, Japan) was used to perform the PCR process. Target genes were amplified using primers; sequences were as follows:

miR-223-F: 5′-TGTCAGTTTGTCAAATA-3′;
miR-223-R: 5′-GTGCAGGGTCCGAGGT-3′;
U6-F: 5′CGCTTCGGCAGCACATATAC-3′;
U6-R: 5′- AAATATGGAACGCT-TCACGA-3′;
RhoB-F:5′-TCGTGTTCAGTAAGGACGAG-3′;
RhoB-R:5′-ACTTCTCGGGGATGTTCTC-3′;
GAPDH-F: 5′- TGAGGCCGGTGCTGAGTATGT -3′;
GAPDH-R: 5′- CAGTCTTCTGGGTGGCAGTGAT-3′;

Relative micro-RNA and mRNA expression level was calculated and recorded by a SDS software (Applied Biosystems, HT7900, Foster City, CA, USA)

### 2.6. Western Blot Analysis

The operation steps of Western blotting are consistent with the description in our previous study. In short, the treated cells were collected 24 h after stretch injury, and then the protein was extracted in a protein extraction kit. After being diluted to equal concentration, the protein samples can be used for electrophoresis. SDS PAGE gel was prepared before electrophoresis, and the same amount of protein was added to each resolving gel, and the process was carried out under steady flow and steady pressure, respectively, and then transferred to the PVDF membrane for sealing. The primary antibody of anti-ZO-1 (1:500; Cell Signaling Technology, Beverly, MA, USA), Caspase-3,cleaved-caspase3, BAX (1:500; Cell Signaling Technology, Beverly, MA, USA), and β-tubulin and GAPDH (1:1000; Santa Cruz Technology, Santa Cruz, CA, USA) was incubated at 4 °C overnight. After cleaning, the second antibody was selected to incubate at room temperature. The PVDF film incubated with antibodies was detected by chemiluminescence. The fluorescence intensity was calculated and evaluated by gel Pro software.

### 2.7. Flow Cytometry

Flow cytometry can be used to examine apoptosis. In our study, bEnd.3 cells cultured in a six-well plate were subjected to stretch injury. After injury, they were cultured for 6 h (37 °C, 5% CO_2_), and then the cells were digested and collected. Annexin V, Alexa fluor 488, and propidium iodide (PI; component B) were mixed in 5 μL solution and further diluted to 100 μL. Then, the cell suspension to be tested was mixed with the reagent and cultured at room temperature in the dark for 10 min. The fully reacted binding solution was added to a 96-well plate. Finally, apoptotic cells were detected by flow cytometry, and the ratio of stained cells to control cells was analyzed by Accuri C6 flow cytometer (BD Biosciences).

### 2.8. Statistical Analysis

All data are presented as the mean ± SD. Equality of variance was assessed by Levene’s test. T-tests were used to compare means between groups. *p* values of <0.05 were considered to indicate statistical significance. Graphic representations of the data were produced using Graph Pad Prism 6 (GraphPad Software, San Diego, CA, USA), and SPSS 20.0 for Windows (SPSS Inc., Chicago, IL, USA) was used for statistical analyses.

## 3. Result

### 3.1. The Expression of miR-223 Increased after SI

**Figure 1:**
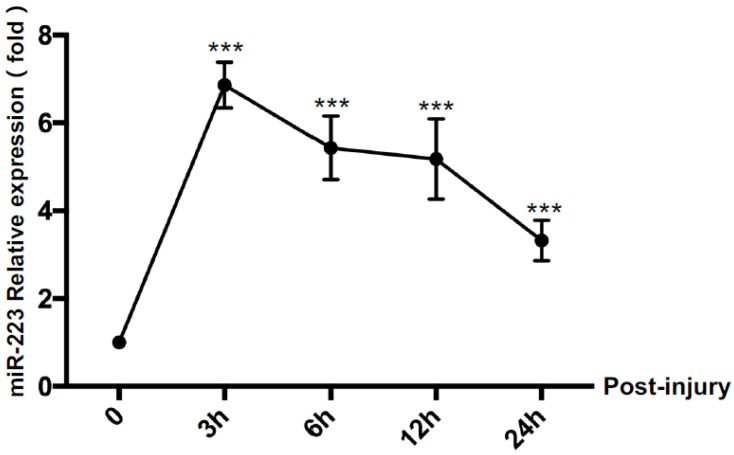
The altered miR-223 level in bEnd.3 cells after SI. The level of miR-223 increased significantly at 3 h, 6 h, 12 h, and 48 h post-injury compared with that at 0 h. *n* = 6 per group; data are presented as mean ± SD; *** *p* < 0.001. miR-223: microRNA-223; SI: stretch injury; bEnd.3: brain-derived endothelial cells. 3; h: hour.

In order to study the expression of miR-223 in BMEC cells after cell injury, the cultured bEnd.3 cells were subjected to SI, and the expression of miR-223 was examined by PCR. At 3, 6, 12, and 24 h after SI, cells were collected, and microRNA was extracted. As shown in Figure 1, the expression of miR-223 markedly increased after SI (*p* < 0.05 vs. control group), and the expression of miR-223 increased to the highest level 3 h after injury.

### 3.2. miR-223 Was Overexpressed in bEnd.3 Cells by Lentivirus Transfection

**Figure 2:**
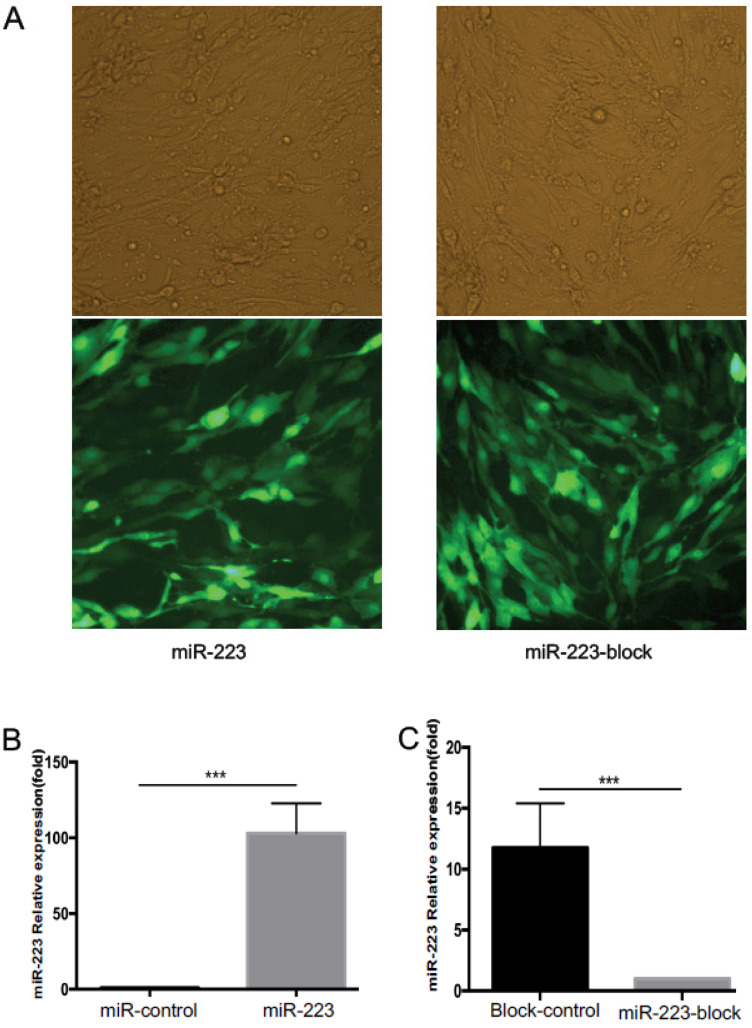
Transfection efficiency and miR-223 expression after transfected with miR-223 and miR-223-block lentivirus. (**A**) Fluorescent cells that transfected with miR-223 and miR-223-block lentivirus. Note that almost all cells were successfully transfected. Scale bar = 30 μm. Bar graph showing a quantification analysis of miR-223 level in miR-223 (**B**) and miR-223-block (**C**) group, which suggested that the expression of miR-223 was significantly altered after transfection with target lentivirus *n* = 8 per group; data are presented as mean ± SD; *** *p* < 0.001. miR-223: microRNA-223; SI: stretch injury.

In order to further study the relationship between miR-223 expression and BMECs, we transfected bEnd.3 cells with lentivirus. As shown in Figure 2A, under the fluorescence microscope, almost all bEnd.3 cells showed green fluorescence, indicating that the transfection was successful. After digesting the cells, we extracted microRNA. PCR results showed the expression of miR-223 in bEnd.3 cells transfected by miR-223 lentivirus was 100 times higher than that in the control group, and the expression of miR-223 in bEnd.3 cells transfected by miR-223-block lentivirus was more than 10 times lower than that in the control group (Figure 2B,C), and this means the expression of miR-223 can be fully regulated (*p* < 0.05 vs. control group).

### 3.3. miR-223 Attenuates the Loss of Tight Junction Protein after SI in bEnd.3 Cells

**Figure 3:**
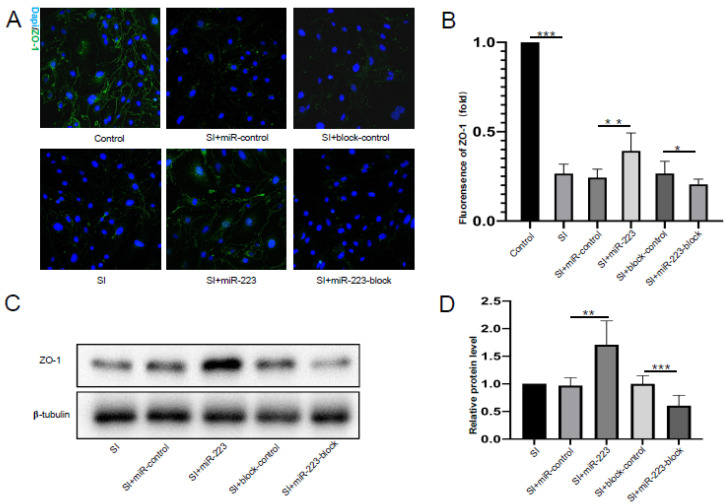
MiR-223 rescued the decrease in tight junction proteins in bEnd.3 cells after SI. (**A**) Immunocytochemistry from fixed bEnd.3 cells 24 h after SI. Note that miR-223 overexpressed bEnd.3 cells have higher fluorescence intensity compared with control group. Scale bar = 30 mm. (**B**) Bar graphs showing the fluorescence of ZO-1. (**C**) Representative results of ZO-1 expression 24 h after stretch injury. (**D**) Bar graphs showing the quantification of ZO-1 expression. *n* = 6 per group; data are presented as the mean ± SD; * *p* < 0.05, ** *p* < 0.01, *** *p* < 0.001, SI versus Control group, miR-223 versus miR-control group, miR-223-block versus block-control group. miR-223: microRNA-223; h: hour; ZO-1: zonula occludens 1; SI: stretch injury.

In order to study the protective effect of miR-223 on the tight junction protein of BMECs, we detected the expression of ZO-1 protein in bEnd.3 cells. The immunofluorescence staining test showed that ZO-1 protein was mainly expressed at the junction of cells. After stretch injury, the expression of ZO-1 in bEnd.3 cells decreased significantly, and compared with the control group, the fluorescence of the miR-223 overexpressed group was significantly enhanced, while the fluorescence of the anti-miR-223 group was significantly decreased (Figure 3A,B). As shown in Figure 3C,D, Western blot tests showed the expression of ZO-1 in bEnd.3 cells with miR-223 overexpression increased significantly after SI, while the expression of ZO-1 decreased significantly after miR-223 block treatment (*p* < 0.05 vs. control group). This suggests that miR-223 has a protective effect on tight junction protein.

### 3.4. miR-223 Inhibits Apoptosis of bEnd.3 Cells after Stretch Injury

**Figure 4:**
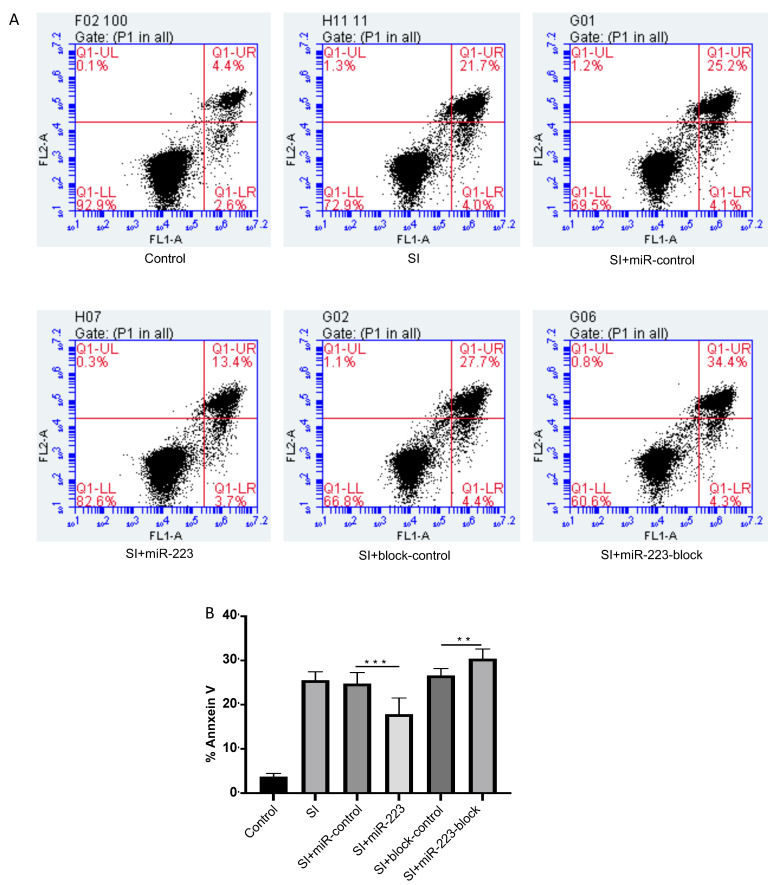
MiR-223 attenuates apoptosis in bEnd.3 cells after SI. BEnd.3 cells were detected by flow cytometry after immunostained with Annexin. (**A**) MiR-223 overexpressed bEnd.3 cells have lower fluorescence intensity compared to cells in miR-control group at 6 h post-injury. MiR-223-block transfected group has higher fluorescence than block-control group. (**B**) Bar graphs showing the quantification of the Annexin V intensity of cells in different groups. *n* = 8 per group; data are presented as the mean ± SD; ** *p* < 0.01, *** *p* < 0.001, miR-223 versus miR-control group, miR-223-block versus block-control group. miR-223: microRNA-223; h: hour; ZO-1: zonula occludens 1; SI: stretch injury.

Flow cytometry-based assays can effectively detect the early stage apoptosis rate of many cells. As shown in Figure 4A,B, the annexin V positive rate of bEnd.3 cells transfected with miR-223 was significantly lower than that of the control group at 6 h post-injury, while the annexin V positive rate of bEnd.3 cells transfected with miR-223-block was significantly higher than that of the control group. These experimental results suggested that miR-223 could inhibit the apoptosis of bEnd.3 cells, which is induced by SI, suggesting that miR-223 has a protective effect on BMECs.

### 3.5. miR-223 Modulated SI-Induced Caspase-3 Activation

**Figure 5:**
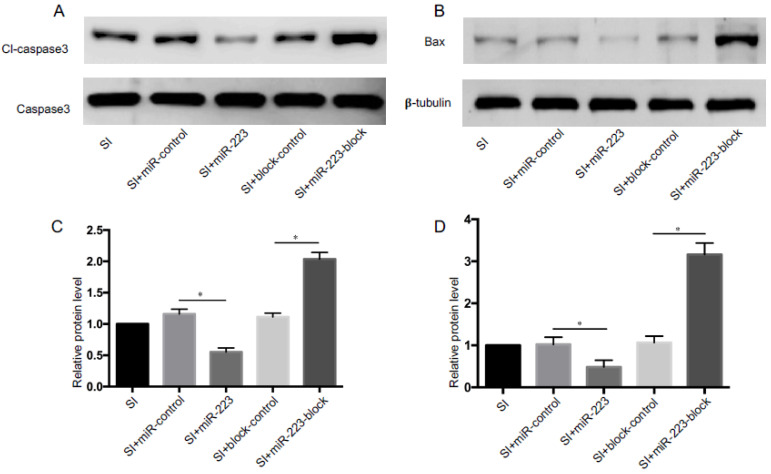
Effects of miR-223 on pro-apoptotic proteins in bEnd.3 cells after SI. Protein expression of cl-caspase-3/caspase-3 (**A**) and BAX (**B**) in Western blots. Bar graphs showing quantification of cl-caspase-3 (**C**) and BAX (**D**), *n* = 6 per group; data are presented as the mean ± SD; * *p* < 0.05, miR-223 versus miR-ontrol group, miR-223-block versus block-control group. miR-223: microRNA-223; cl-caspase-3: cleaved-caspase-3; SI: stretch injury.

Caspase-3 activation is an important step in the initiation of apoptosis, and the expression level of pro-apoptotic proteins (Bax and cl-caspase-3) reflects the degree of apoptosis. Western blot results showed that the expression of cl-caspase-3/caspase-3 and Bax increased in bEnd.3 cells after SI (Figure 5A,B). At the same time, we found that the expression of cl-caspase-3 and Bax decreased significantly in miR-223 transfected cells (Figure 5C); nevertheless, their expression was higher in the miR-223-block group (*p* < 0.05 vs. control group). These results confirmed the inhibitory effect of miR-223 on apoptosis in bEnd.3 cells.

### 3.6. MiR-223 Inhibited RhoB Expression, and Knockdown of RhoB Decreased Caspase-3 Activation

**Figure 6:**
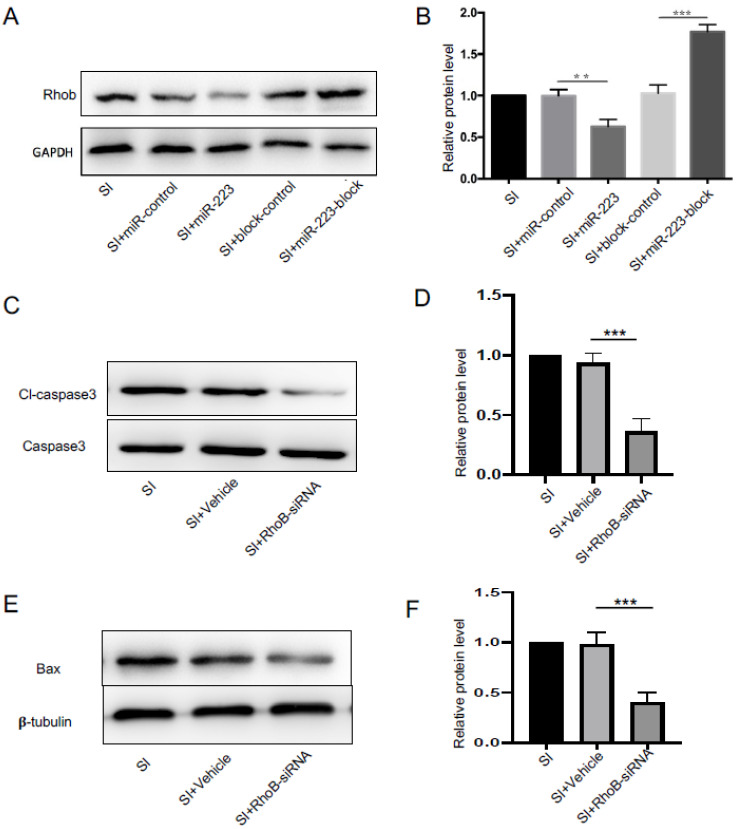
MiR-223 inhibited rhoB expression, and knockdown of RhoB decreased caspase-3 activation. (**A**) Protein expression of RohB in Western blots. (**B**) Bar graphs showing quantification of RohB. (**C**) RohB-siRNA limited the expression of cl-caspase-3 in Western blots. (**D**) Bar graphs showing quantification of cl-caspase-3. (**E**) RohB-siRNA limited the expression of BAX in Western blots. (**F**) Bar graphs showing quantification of BAX. *n* = 6 per group; data are presented as the mean ± SD; ** *p* < 0.01, *** *p* < 0.001, miR-223 versus miR-control group, miR-223-block versus block-control group, siRNA versus vehicle group. miR-223: microRNA-223; cl-caspase-3: cleaved-caspase-3.

To explore the molecular mechanism of the anti-apoptosis effect of miR-223, We detected the relationship between miR-223 and RhoB protein expression. Western blot results showed that the expression of RhoB in bEnd.3 cells was decreased after transfection with miR-223, But as shown in Figure 6A,B, when we did block miR-223, we got the opposite result (*p* < 0.05 vs. control group).

In order to further investigate the role of RhoB in the anti-apoptosis effect of miR-223, we used siRNA to inhibit the expression of RhoB. The results showed that the addition of RhoB-siRNA could significantly reduce the expression of cl-caspase3 and Bax (Figure 6C–F) in bEnd.3 cell after SI (*p* < 0.05 vs. vehicle group). The above results suggest that miR-223 may inhibit the occurrence of apoptosis by the inhibition of RhoB expression.

## 4. Discussion

In recent years, the study of miR-223 in the field of brain and spinal injury has attracted researchers’ attention. MiR-223 has been proven to inhibit neuronal apoptosis and reduce neuroinflammation [5,6], and the transplantation of neural stem cells with overexpression of miR-223 showed repair effects in spinal cord injury, whereas the relationship between miR-223 and BMECs or BBB is still lacking. In our study, we demonstrated the anti-apoptosis effect of miR-223. SI device and bEnd.3 cells were chosen for simulating the damage of BMECs in TBI, and via lentivirus transfection, we regulated the expression of miR-223. Through our experiments, we revealed that the overexpression of miR-223 can inhibit the apoptosis of bEnd.3 cells, and this may be owing to the inhibition of RhoB.

As major components of BBB, BMECs and tight junction proteins (among BMECs) play critical roles in maintaining the integrity of BBB. Therefore, BMECs are often chosen as major experimental cells to study BBB. Zhang demonstrated that lncRNA DANCR maintained BBB integrity by regulating Mir-33a-5p/XBP1s through in vitro experiment of BMECs [20]. URB597 and Andrographolide were found to reduce the apoptosis and permeability damage of BMECs caused by oxygen-glucose deprivation [21]. Li performed a glucose and oxygen deprivation experiment on human BMECs and demonstrated the BBB protective effect of CIRC_0006768 [22]. Sun found that donepezil ameliorated oxygen-glucose deprivation/reoxygenation-induced BMEC dysfunction and indicated that Donepezil may have a BBB-protective effect [23]. In our study, bEnd.3 cells were used for in vitro experiment. We found that overexpression of miR-223 could attenuate SI-induced apoptosis of bEnd.3 cells, and the result indicated that miR-223 may play a role in maintaining the integrity of BBB.

MiR-223 is a miRNA with important functions and has been widely investigated in recent years. It was reported to play regulatory roles in hematopoiesis, carcinogenesis, inflammation, and lipid metabolism [2]. Moreover, previous studies have demonstrated that microarray and RT-qPCR analysis showed a (4.7–160)-fold increase in the brain tissue of rats after TBI injury [7,8,9]. However, there are only a limited number of studies focused on its role in the central nervous system, and little literature is about the relation between miR-223, BMECs, and BBB. Wang found that upregulation of miR-223 inhibits the apoptosis of hippocampal neurons induced by convulsion and thus alleviates brain injury [5]. MiR-223/TIAL1 is also involved in the neuroprotective effects of dexmedetomidine [24]. MiR-223 has been shown to inhibit the inflammatory response in a mouse model of intracerebral hematoma [6]. In the study of glioma, it was found that overexpression of miR-223 can inhibit the proliferation and migration of glioma cells [25]. In addition, expression of miR-223 increased after spinal cord injury in rats, and transplantation of neural stem cells overexpressing miR-223 helped to repair spinal cord injury [26]. In our study, we found that the expression of miR-223 was significantly increased after SI, which was consistent with previous literature reports [7,8,9]. In the miR-223 overexpression group, the expression of tight junction protein ZO-1 of bEnd.3 cells was significantly higher than that in the control group after SI. Through flow cytometry, we found that miR-223 could inhibit the apoptosis of bEnd.3 cells. This suggests that miR-223 has a protective effect on BMECs, and through animal experiments in our follow-up study, it may be demonstrated that miR-223 plays a role in maintaining the integrity of BBB. This also suggests that the role of miR-223 in the nervous system deserves more research.

RhoB plays an important role in the regulation of apoptosis. Xiao showed that RhoB knockdown attenuated the apoptosis of SH-SY5Y cells [27]. Moreover, the miR-223-RhoB signaling pathway has become a proven regulatory pathway for apoptosis. Inhibition of miR-223 led to upregulation of RhoB and, in turn, promoted apoptosis of Colorectal cancer cells [28]. Additionally, miR-223–3p could regulate the cellular apoptosis of non-small cell lung cancer by targeting RhoB [29]. Through cell experiments, we found that the expression of RhoB was inhibited in bEnd.3 cells were transfected with miR-223 overexpression virus. In order to explore the relationship between the anti-apoptotic effect and the RhoB gene, we added RhoB-siRNA and found that it could inhibit the apoptosis of bEnd.3. Therefore, we inferred that the anti-apoptosis effect of miR-223 is at least partially realized by inhibiting the expression of RhoB. Based on these experimental results, we believe that RhoB can be used as a gene target, and perhaps the apoptosis of BMECs may be reduced through regulation of RhoB expression so as to ameliorate the leakage of BBB.

There are still limitations in our study. The in vitro study of BMECs is not equivalent to the in vivo study of BBB, so it is not yet completely proven that miR-223 has a protective effect on BBB. Future studies may include animal experiments to translationally examine the effect of miR-223 on the permeability of BBB in vivo.

## 5. Conclusions

The results of this study suggest that miR-223 can alleviate the apoptosis of BMECs induced by SI, and this anti-apoptotic effect is at least partially achieved by inhibiting the expression of RhoB. Additionally, miR-223 may play a role in maintaining the integrity of BBB during TBI.

## Data Availability

The raw/processed data required to reproduce these findings cannot beshared at this time as the data also forms part of an ongoing study.
